# Opium Antidotes Exposed

**Published:** 1876-10

**Authors:** 


### "Opium Antidotes," Exposed.

In the month of August last, Dr. George F. French, of Portland, Maine, was applied to by an opium-eater who asked his advice about a preparation advertised as a sure cure for the opium habit. Naturally being suspicious of such an article, he sent to the manufacturer, Mrs. J. A. Drollinger, of La Porte, Indiana, for a sample bottle. This was furnished, but, as we understand, the proprietress declined to give any information as to its composition, saying, however, that it "is harmless when taken as directed," and "does not contain opium in any form." Failing to be satisfied with this assertion, the doctor applied such chemical tests as he conveniently could and got the reactions of morphia. But to make assurance doubly sure, and to supplement and confirm the chemical test by a physiological one, he secretly administered a small dose of the "antidote" to a person who had a peculiar idiosyncrasy with reference to opium. The speedy result was as has been anticipated, a manifestation of the symptoms which in this individual had always followed the exhibition of opium, namely, suffusion of the eyes, loss of voice, pain in the head, and insomnia. Dr. French then reported these facts to the Cumberland County Medical Society, which, at his suggestion, at once appointed a committee to further investigate the matter, and, and voted to bear the expense of whatever analyses might be necessary.

At the regular meeting of the society in September the committee presented the following

REPORT.

The committee to whom was assigued the duty of investigating the so-called "opium antidote" prepared by Mrs. J. A. Drollinger, of La Porte, Indiana, beg leave to report that a sample bottle of the article, which was obtained directly from the manufacturer, was sent to Dr. Edward R. Squibb, of Brooklyn, N. Y., for quantitative analysis. His onorous engagements rendered it impossible for him to conduct the investigation in person, but he sent the specimen to Messrs. Walz and Stillwell, chemists, New Yotk, a firm which he thoroughly confided in and endorses. So deeply interested did he become in the project that he insisted upon bearing the expense of the analysis, in spite of the committee's expressed unwillingness to have him assume such a tax.

Walz and Stillwell report that "this sample is glycerine colored with analine red, and containing in solution crystalized sulphate of morphia 1.383 per cent. by weight,"—about seven grains to the ounce.

While this investigation was progressing, the committee found another alleged "opium antidote," prepared by "Dr. S. B. Collins, the Great Narcologist of the Age," likewise of La Porte, Indiana. A specimen of this was submitted to Dr. Henry Carmichael, Professor of Chemistry in Bowdoin College and Assayer of the State of Maine, who arrived at the following conclusions :—

"(1.) The opium antidote contains morphine.

"(2.) The morphine is combined with sulphuric acid.

"(3.) The sulphate of morphine amounts to 3.2 per cent., or fourteen grains to the ounce."

Dr. Walz says that he made an anlysis of Collin's "Antidote" in 1871, and found that it contained morphia, though he did not ascertain the quantity.

In conclusion, your committee respectfully suggest that the society take some action which will result in the wide dissemination of the information which has been acquired concerning these dangerous preparations.

Frederick Henry Gerrish,

George F. French,

Thomas A. Foster. } *Committee.*

The society instructed the committee to present their report to some prominent medical journal, and if it seemed to them advisable, to give the public warning of the danger to which it is exposed through the newspapers of the State. A vote of thanks was passed to Dr. Squibb for his generous assistance.

The importance of this exposure is too obvious to require any extensive comment on our part. Physicians now have something better than general reasons to offer their patients when warning them to shun such nostrums. The profession will not be insensible to the valuable services which the Cumberland County Medical Society has rendered it and the community, and it is to be hoped that other similar bodies will bc encouraged to display equal enterprise and spirit. There is a great opportunity for our brethren in the region of La Porte to distinguish themselves as guardians of the health of the people, and we trust that they will not be slow to follow up the track so well opened by their fellows in Maine.—*Boston Medical and Surgical Journal.*

